# Supplementary material for: Small molecule degraders of the hepatitis C virus protease reduce susceptibility to resistance mutations
Source: Nat Commun. 2019 Aug 1;10:3468. doi: 10.1038/s41467-019-11429-w (PMC6672008; doi:10.1038/s41467-019-11429-w)
Supplement: Supplementary file 1 — Supplementary Information [file 41467_2019_11429_MOESM1_ESM.pdf]

Supplementary Information

Small Molecule Degraders of the Hepatitis C Virus Protease Reduce Susceptibility to Resistance Mutations

Authors:

Mélissanne de Wispelaere[1‡], Guangyan Du[2,3‡], Katherine A. Donovan[2,3], Tinghu Zhang[2,3], Nicholas A. Eleuteri[3], Jingting C. Yuan[3], Joann Kalabathula[3], Radosław P. Nowak[2,3], Eric S. Fischer[2,3], Nathanael S. Gray[2,3], Priscilla L. Yang[1*]

Affiliations:

[1] Department of Microbiology and Blavatnik Institute, Harvard Medical School, Boston, Massachusetts, USA

[2] Department of Biological Chemistry and Molecular Pharmacology, Harvard Medical School, Boston, Massachusetts, USA

[3] Department of Cancer Biology, Dana-Farber Cancer Institute, Boston, Massachusetts, USA

[‡] Contributed equally

* Lead Contact and corresponding author. Email: priscilla_yang@hms.harvard.edu

SUPPLEMENTARY FIGURES

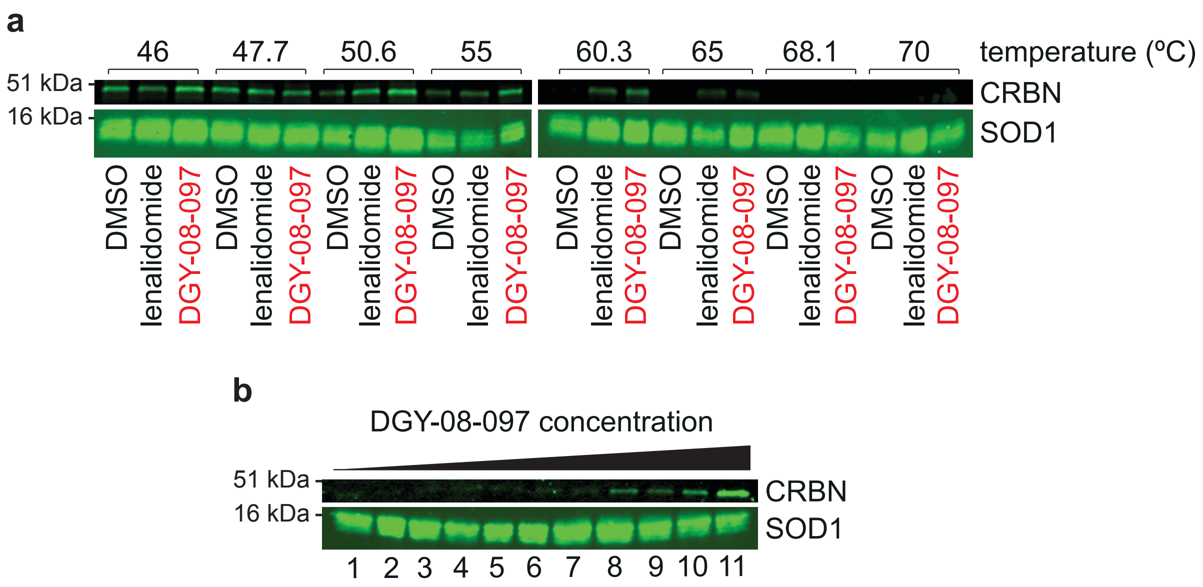

Supplementary Figure 1. Assessment of CRBN engagement using a cellular thermal shift assay.

a Cellular thermal shift assay for DGY-08-097 stabilization of CRBN. Thermal denaturation curves (46 ºC – 70 ºC) and Western blotting analysis of CRBN and thermally stable control SOD1 after treatment with 10 µM lenalidomide, DGY-08-097, or DMSO control. Source data are provided as a Source Data file.

b Cellular thermal dose-response assay for DGY-08-097 stabilization of CRBN. Dose-response curves (10 nM – 50 µM) and Western blotting analysis demonstrating the concentration-dependent stabilization of CRBN treated with DGY-08-097 at 61 ºC. Source data are provided as a Source Data file.

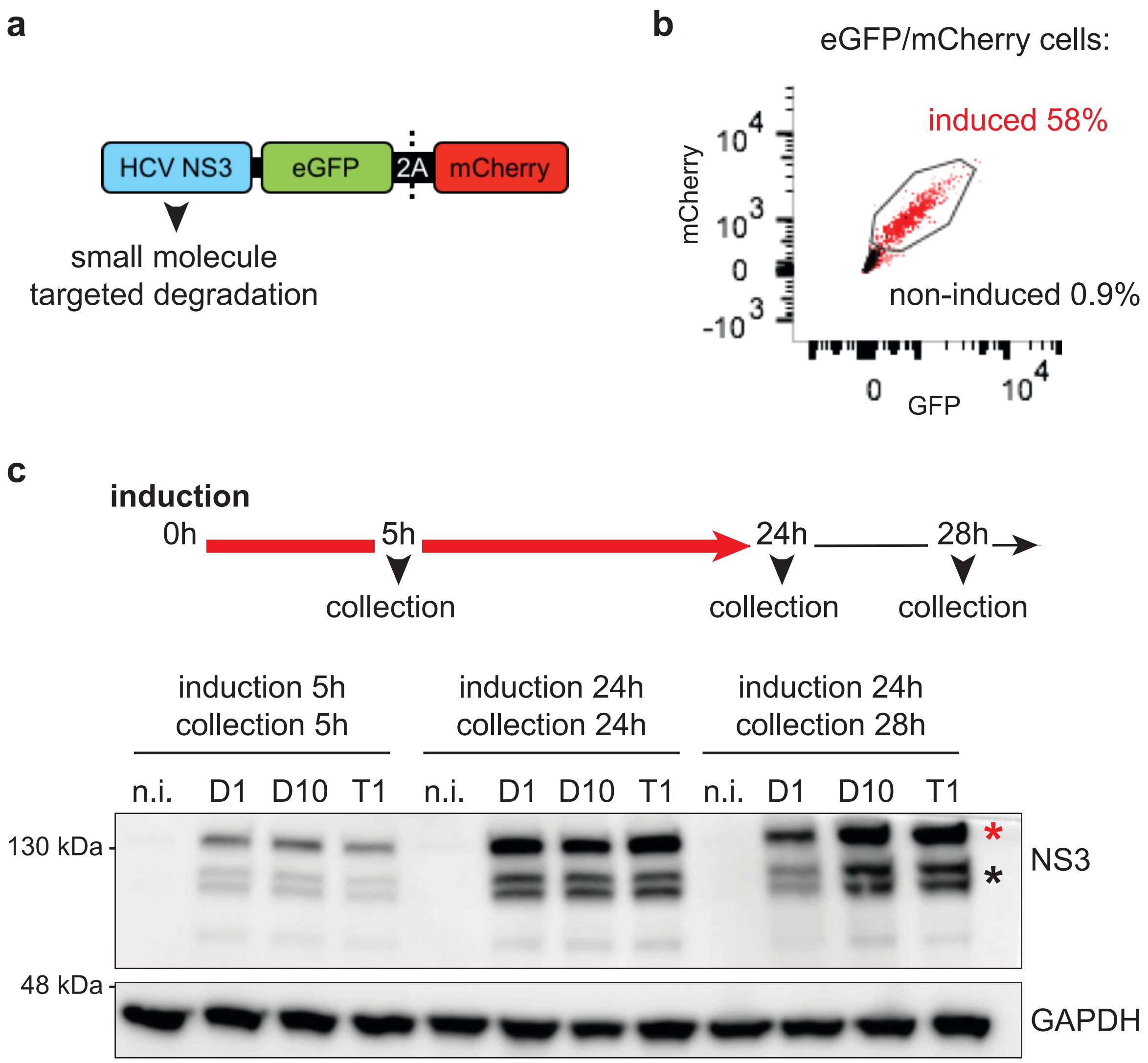

Supplementary Figure 2. Characterization of the cell line used to evaluate cellular degradation of HCV NS3.

a Schematic depiction of the model NS3 substrate protein used to evaluate NS3 degraders. We designed a reporter construct in which the HCV NS3 protein is fused to eGFP, which is separated from the mCherry protein by the FMDV 2A ribosomal skipping sequence. eGFP fluorescence serves as a proxy for the abundance of the HCV NS3-eGFP protein in the cells, while mCherry fluorescence serves as a control for expression of the model substrate. The HCV protease NS4A cofactor is not expressed in the cells, and thus the NS3 protein does not have protease activity in this system. We established a stable 293 clonal cell lines that expresses the HCV NS3-eGFP and mCherry reporter under the control of the tetracycline-responsive promoter.

b Characterization of HCV NS3-eGFP and mCherry expression by flow cytometry. Cells were induced for 24 hours with 1 μg.mL^{-1} tetracycline, and the number of cells expressing eGFP and mCherry was quantified by flow cytometry. Non-induced cells served as controls. The scatter plot represents the distribution of the cell population based on their levels of eGFP (*x* axis) and mCherry (*y* axis) fluorescence. The proportion of induced cells expressing the fusion protein varied across experiments and was comprised of 55 to 85% of the total cell population. A representative experiment out of $n > 3$ independent experiments is shown.

c Characterization of HCV NS3-eGFP fusion protein expression by Western blotting. Cells were induced for 5 hours or 24 hours with doxycycline at 1 μg.mL^{-1} (D1) or 10 μg.mL^{-1} (D10), or tetracycline at 1 μg.mL^{-1} (T1). Non-induced cells (n.i.) served as controls. Cells were collected at various times post-induction and protein expression was analyzed by Western blotting using an antibody that detects HCV NS3 protein. We could detect accumulation of the full-length fusion protein (124 kDa, indicated by a red star) and of the HCV NS3-eGFP fusion product (95 kDa, indicated by a black star; the protein often migrated as a doublet). Source data are provided as a Source Data file.

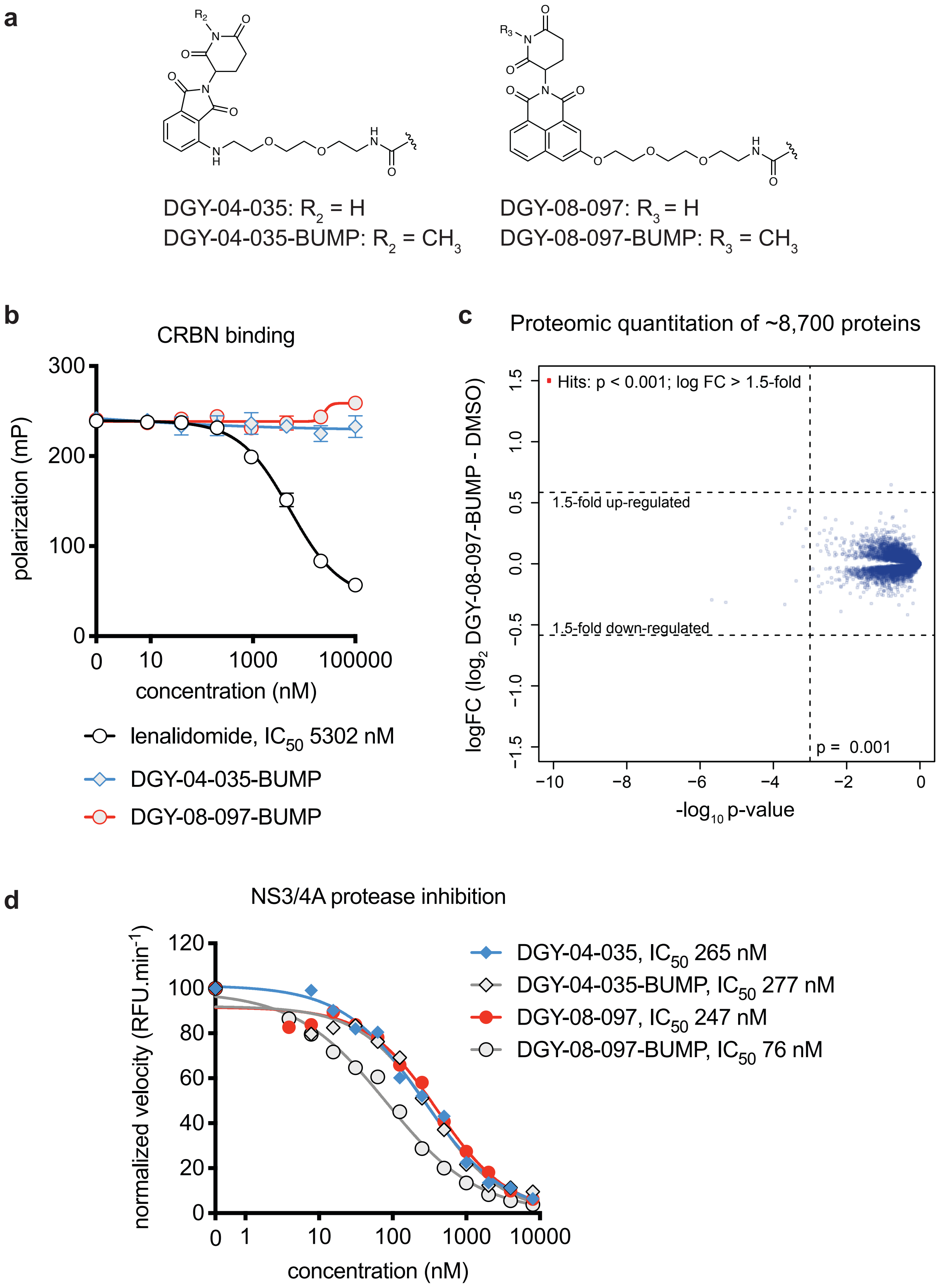

Supplementary Figure 3. Characterization of negative control compounds.

a Structures of DGY-04-035-BUMP and DGY-08-097-BUMP. Structures of degraders DGY-04-035 and DGY-08-097 and their respective negative controls are reproduced here from Figure 1.

b Competitive binding assay to DDB1ΔB-CRBN[1]. Increasing concentrations of compounds were titrated to preformed DDB1ΔB-CRBN-lenalidomideAtto565 complex. Data are presented as means normalized to DMSO ± standard deviation of *n* = 4 technical replicates. Neither DGY-04-035-BUMP nor DGY-08-097-BUMP exhibit detectable binding to CRBN. mP: millipolarization units.

c Quantitative proteomics analysis of NS3-eGFP expressing 293 cells treated with the DGY-08-097-BUMP or DMSO control. The scatter plot depicts the $\log_2$ fold change (FC) in protein abundance in induced cells treated for 4 hours with 1000 nM DGY-08-097-BUMP compared to the DMSO control. Data shown are of a single quantitative TMT 10-plex experiment (showing ~8,700 proteins, each quantified by ≥ 2 unique peptides). Significant changes were assessed by a moderated t-test as implemented in the limma package[2]. The $\log_2$ fold change is shown on the *y*-axis and negative $\log_{10}$ *P* values on the *x*-axis (*n* = 3 independent biological replicates). No significant degradation of HCV NS3 or any other cellular protein was observed for DGY-08-097-BUMP.

d Measurement of NS3/4A protease inhibition *in vitro*. Cell lysates containing the endogenous NS3/4A protease were prepared from cells stably expressing an HCV subgenomic replicon. The cell lysates were preincubated with candidate degraders present at a range of concentrations, and then incubated with the HCV NS3 FRET peptide substrate RET S1. Cleavage of the FRET substrate was measured for 1 hour at 30°C, and the rate of enzymatic cleavage was fitted by linear regression. The concentration that led to a 50% decrease in HCV NS3/4A enzymatic activity (IC_{50}) was determined by nonlinear regression. Data are presented as means normalized to DMSO ± standard deviation of *n* = 2 technical replicates. One representative experiment is shown, with IC_{50} values averaged from *n* ≥ 3. Source data are provided as a Source Data file. RFU: relative fluorescence units.

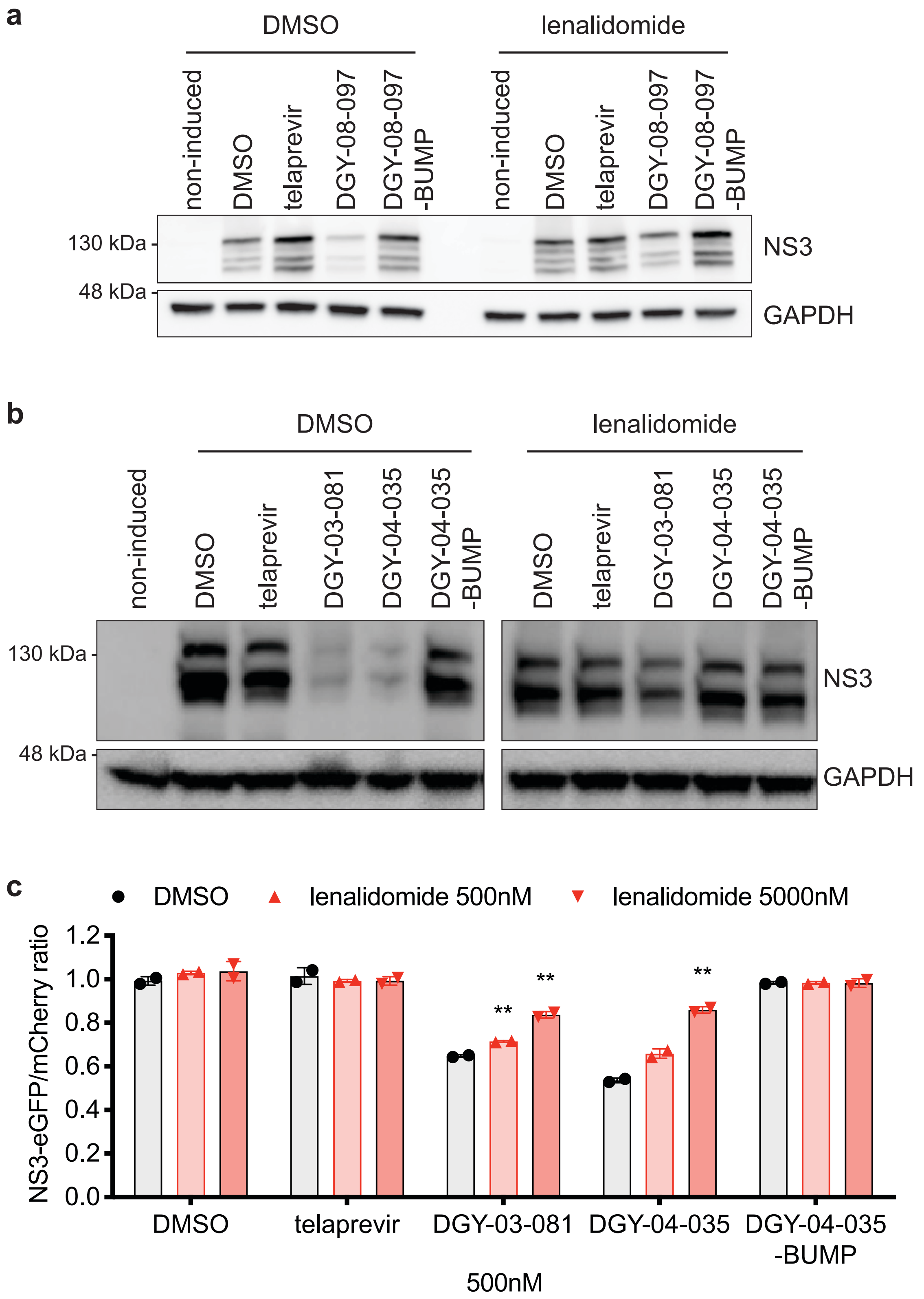

Supplementary Figure 4. The effect of NS3-targeting degraders is reversed in the presence of excess lenalidomide.

a and **b** Expression of NS3-eGFP-2A-mCherry was induced, and cells were treated for 4 hours with 1000 nM of each telaprevir-derived compound and co-treated with either DMSO or 5000 nM lenalidomide. The cells were collected for Western blot analysis of the indicated proteins.

a One representative experiment for DGY-08-097 and DGY-08-097-BUMP is shown from n = 3. Source data are provided as a Source Data file.

b One representative experiment for DGY-03-81, DGY-04-035, and DGY-04-035-BUMP is shown from n = 2. Source data are provided as a Source Data file.

c Expression of NS3-eGFP-2A-mCherry was induced, and cells were treated for 4 hours with 500 nM of each telaprevir-derived compound, and co-treated with either DMSO or the indicated concentration of lenalidomide. The eGFP and mCherry signals were quantified by flow cytometry analysis. Data are presented as means normalized to DMSO ± standard deviation of n = 2 independent experiments. Asterisks indicate that the differences between samples are statistically significant, using the unpaired t-test (**, $0.001 < p < 0.01$; not significant, $p > 0.05$). Source data are provided as a Source Data file.

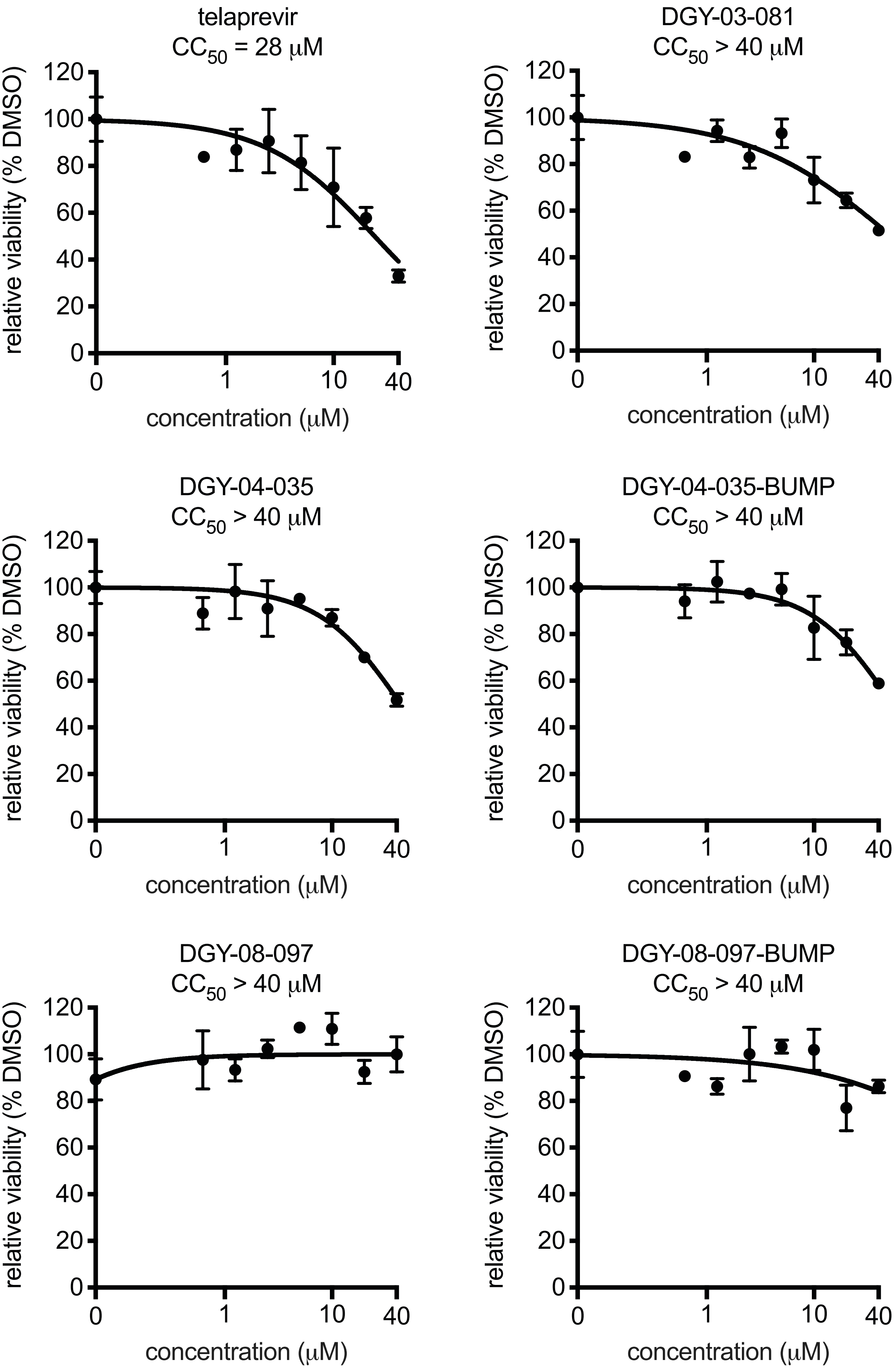

telaprevir
CC50 = 28 μM
DGY-03-081
CC50 > 40 μM
DGY-04-035
CC50 > 40 μM
DGY-04-035-BUMP
CC50 > 40 μM
DGY-08-097
CC50 > 40 μM
DGY-08-097-BUMP
CC50 > 40 μM
relative viability (% DMSO)
0
20
40
60
80
100
120
concentration (μM)
0
1
10
40

Supplementary Figure 5. The NS3-targeting degraders are non-cytotoxic at the concentrations at which degradation of NS3 and antiviral activity are observed. Huh7.5 cells stably expressing an HCV subgenomic replicon were treated with the NS3 degraders at a range of concentrations. Cell viability was measured 24 hours later, and data are presented as means normalized to DMSO ± standard deviation of *n* = 3 experimental replicates. CC_{50} values, corresponding to the concentrations of small molecule that caused 50% loss of viability, were determined by non-linear regression. One representative experiment is shown, with CC_{50} values averaged from *n* = 2. Source data are provided as a Source Data file.

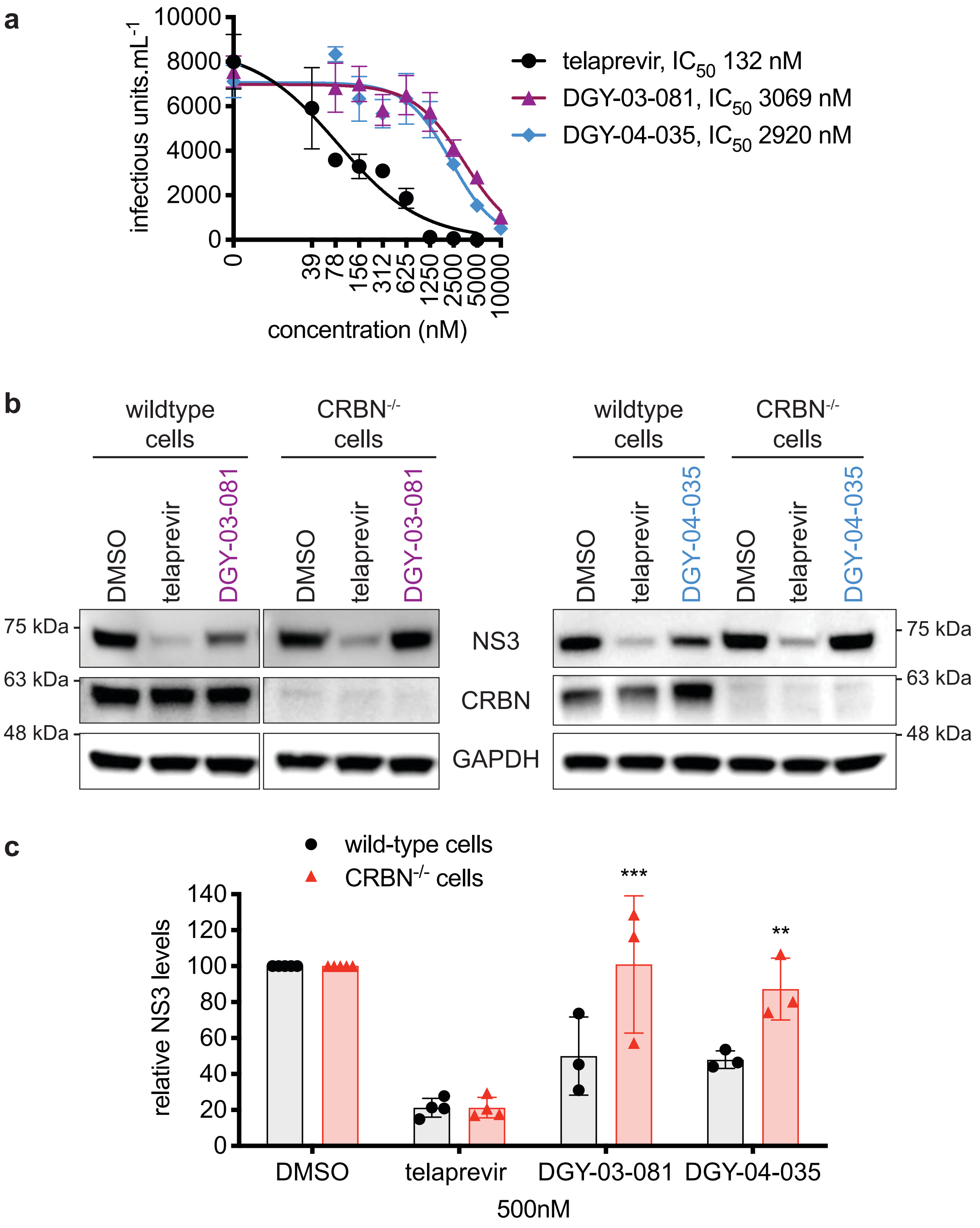

Supplementary Figure 6. Characterization of CRBN dependence for antiviral activity of the NS3-targeting degraders.

a Evaluation of antiviral activity in a cellular infectious assay. Huh7.5 cells were infected with HCV-JFH1-ad at a multiplicity of infection (MOI) of 0.1. The infected

cells were treated from 24 to 48 hours post-infection with NS3 degraders at a range of concentrations. The amount of infectious virus released to the supernatants at 48 hours post-infection was measured using a 50% tissue infectious dose ($TCID_{50}$) assay. The concentration of compound that led to a 50% reduction in viral titers (IC_{50}) was determined by non-linear regression. Data are presented as means ± standard error of n = 4 technical replicates. One representative experiment is shown, with IC_{50} values averaged from $n \geq 2$ independent experiments. Source data are provided as a Source Data file.

b and **c** Degradation of NS3 contributes to the antiviral activity of the NS3 degraders. Wildtype Huh7.5 and Huh7.5 $CRBN^{-/-}$ cells were infected with HCV-JFH1-ad at a MOI of 0.1. The infected cells were treated from 24 to 48 hours post-infection with a 1000 nM of each indicated small molecule concentrations. Chemical structures for DGY-03-081 and DGY-04-035 are shown.

b The abundance of HCV NS3, CRBN and GAPDH in cells was determined by Western blot analysis of cell lysates. Source data are provided as a Source Data file. One representative experiment is shown from n = 3 independent experiments.

c The abundance of NS3 was normalized to the band intensity of the loading control (GAPDH) and is reported as a percentage of the DMSO-treated control. Data are presented as means ± standard error of n = 3 independent experiments. Asterisks indicate that the differences between samples are statistically significant, using the unpaired t-test (***, $p > 0.001$; **, $0.001 < p < 0.01$; not significant, $p > 0.05$). Source data are provided as a Source Data file.

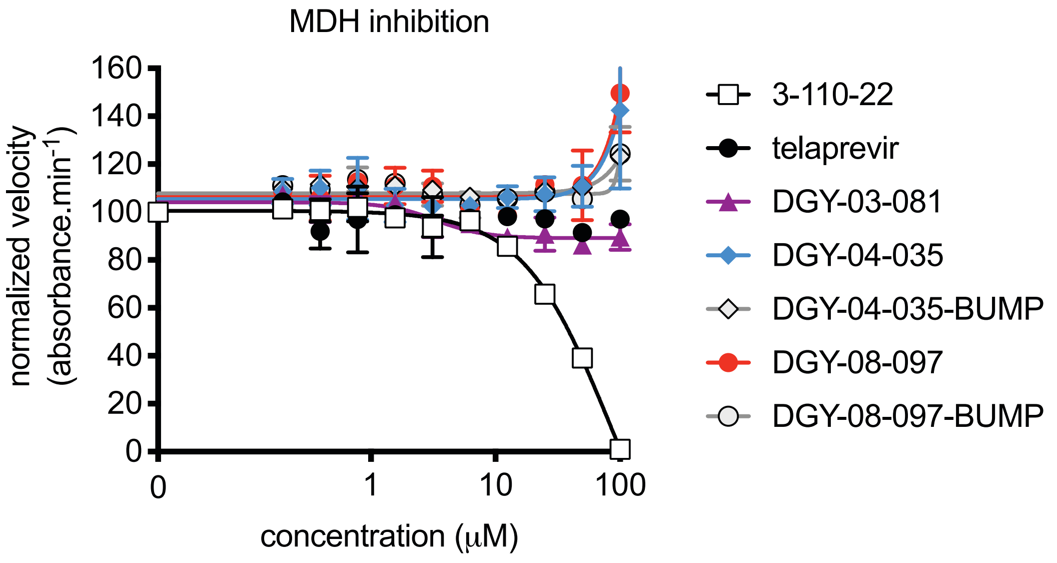

Supplementary Figure 7. Malate dehydrogenase activity assay to detect PAINS activity.

Malate dehydrogenase (MDH) activity was measured for 5 minutes at 25°C, and the rate of enzymatic activity was fitted by linear regression. The activity measured at various compound concentrations was normalized to the activity of the DMSO negative control to derive the percent enzyme activity. Representative data from one of n = 2 independent experiments are presented as means normalized to DMSO ± standard deviation of n = 2 technical replicates.

SUPPLEMENTARY METHODS

Primers used in this study

NotI-HCV-3431-FW: 5’-ATAAGAATGCGGCCGCGCTCCCATCACTGCTTATGCCCA-3’

KpnI-HCV-5323-RV: 5’-GGGGTACCGGGTCATGACCTCAAGGTCAGCT-3’

pcDNA-FRT-TO-GA-FW: 5’-CGTTTAAACCCGCTGATC-3’

pcDNA-FRT-TO-GA-RV: 5’-GATCCGAGCTCGGTACCAAG-3’

HCV-NS3-GA-FW: 5’-CTTGGTACCGAGCTCGGATCCGATATGGCTCCCATCAC-3’

HCV-NS3-GA-RV: 5’-CTGATCAGCGGGTTTAAACGAAACGGGCCCTCTAGACTC-3’

HCV(V2440L)-7643-FW: 5'-GAGGACGATACCACCTTGTGCTGCTCCATGT-3’

HCV(V2440L)-7673-RV: 5'-ACATGGAGCAGCACAAGGTGGTATCGTCCTC-3'

HCV(V55A)-3578-FW: 5'-GGGGTTTTGTGGACTGCATACCACGGAGC-3'

HCV(V55A)-3606-RV: 5'-GCTCCGTGGTATGCAGTCCACAAAACCCC-3'

HCV(A156S)-3880-FW: 5'-CGTTGGGCTCTTCCGAAGTGCTGTGTGCTCTC-3'

HCV(A156S)-3911-RV: 5'-GAGAGCACACAGCACTTCGGAAGAGCCCAACG-3'

HCV-3431-FW: 5’-GCTCCCATCACTGCTTATGCCCA-3’

HCV-5323-RV: 5’-GGTCATGACCTCAAGGTCAGCT-3’

Restrictions sites and mutations are underlined.

DGY-03-081 synthesis and characterization

N^2-((*S*)-1-cyclohexyl-2-((*S*)-1-((1*S*,3a*R*,6a*S*)-1-((*S*)-1-(cyclopropylamino)-1,2-dioxohexan-3-ylcarbamoyl)hexahydrocyclopenta[*c*]pyrrol-2(1*H*)-yl)-3,3-dimethyl-1-oxobutan-2-ylamino)-2-oxoethyl)-*N^5*-(6-(2-(2,6-dioxopiperidin-3-yl)-1-oxoisoindolin-4-ylamino)hexyl)pyrazine-2,5-dicarboxamide (DGY-03-081).

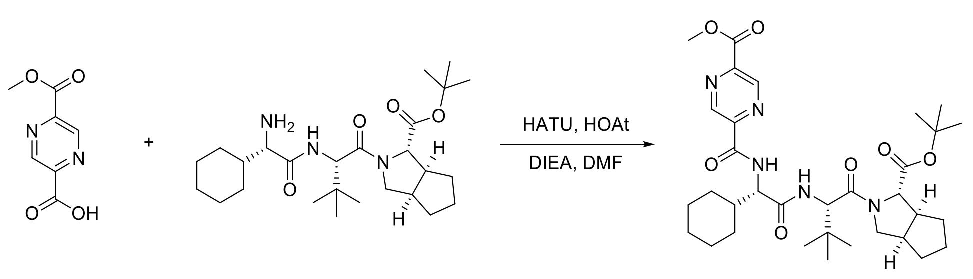

To a solution of 5-(methoxycarbonyl)pyrazine-2-carboxylic acid (208 mg, 1.14 mmol) and (1*S*,3a*R*,6a*S*)-*tert*-butyl 2-((*S*)-2-((*S*)-2-amino-2-cyclohexylacetamido)-3,3-

dimethylbutanoyl)octahydrocyclopenta[*c*]pyrrole-1-carboxylate (530 mg, 1.14 mmol) in DMF (8 mL), DIEA (736 mg, 5.70 mmol), HATU (867 mg, 2.28 mmol) and HOAt (31 mg, 0.228 mmol) were added under ice bath. The reaction was stirred for 10 mins. Quenched the reaction with ice water. The mixture was stirred for 30 mins under ice bath. The white precipitate came out. Filtered the solid and washed with ice water. Dried the solid under vacuum to get (1*S,*3a*R,*6a*S*)-*tert*-butyl 2-((*S*)-2-((*S*)-2-cyclohexyl-2-(5-(methoxycarbonyl)pyrazine-2-carboxamido)acetamido)-3,3-dimethylbutanoyl)octahydrocyclopenta[c]pyrrole-1-carboxylate (560 mg, 0.89 mmol, 78%).
LC/MS *m*/*z* calculated for $[M+H]^+$ 628.4, found 628.4.

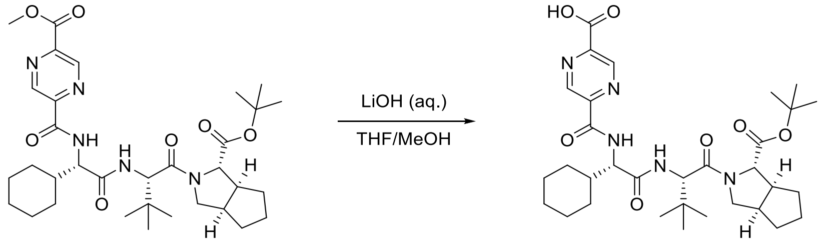

To the solution of (1*S,*3a*R,*6a*S*)-*tert*-butyl 2-((*S*)-2-((*S*)-2-cyclohexyl-2-(5-(methoxycarbonyl)pyrazine-2-carboxamido)acetamido)-3,3-dimethylbutanoyl)octahydrocyclopenta[*c*]pyrrole-1-carboxylate (250 mg, 0.40 mmol) in THF/MeOH (1.6 mL/0.1 mL) LiOH (aq., 0.80 mL, 0.80 mmol, 1M) was added at room temperature. The reaction was stirred overnight. To the mixture, HCl (aq. 2M) was added to acidify the solution to pH = 3. Extracted the mixture with EA (10 mL * 3). The organic layer was combined and evaporated under vacuum to get crude product 5-((*S*)-2-((*S*)-1-((1*S,*3a*R,*6a*S*)-1-(*tert*-butoxycarbonyl)hexahydrocyclopenta[*c*]pyrrol-2(1*H*)-yl)-3,3-dimethyl-1-oxobutan-2-ylamino)-1-cyclohexyl-2-oxoethylcarbamoyl)pyrazine-2-carboxylic acid (223 mg, 0.364 mmol, 91%) without any further purification.
LC/MS *m*/*z* calculated for $[M+H]^+$ 614.3, found 614.3

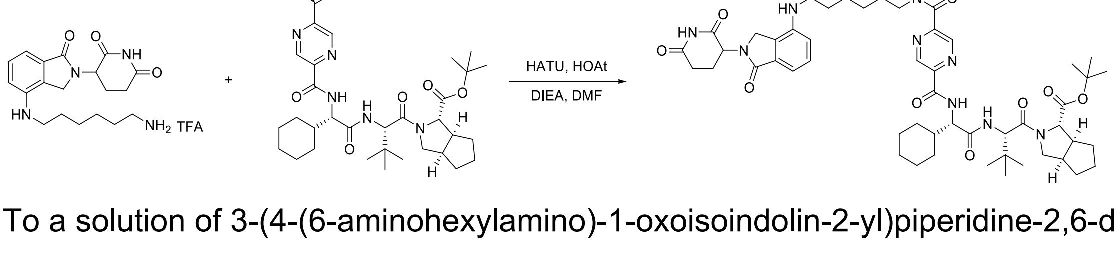

To a solution of 3-(4-(6-aminohexylamino)-1-oxoisoindolin-2-yl)piperidine-2,6-dione

TFA salt (70 mg, 0.15 mmol) and 5-((*S*)-2-((*S*)-1-((1*S,*3a*R,*6a*S*)-1-(*tert*-butoxycarbonyl)hexahydrocyclopenta[*c*]pyrrol-2(1*H*)-yl)-3,3-dimethyl-1-oxobutan-2-ylamino)-1-cyclohexyl-2-oxoethylcarbamoyl)pyrazine-2-carboxylic acid (76 mg, 0.12 mmol) in DMF (1 mL), DIEA (78 mg, 0.60 mmol), HATU (91 mg, 0.24 mmol) and HOAt (3.3 mg, 0.024 mmol) were added under ice bath. The reaction was stirred for 10 mins. The reaction mixture was purified with HPLC to get (1*S,*3a*R,*6a*S*)-*tert*-butyl 2-((2*S*)-2-((2*S*)-2-cyclohexyl-2-(5-(6-(2-(2,6-dioxopiperidin-3-yl)-1-oxoisoindolin-4-ylamino)hexylcarbamoyl)pyrazine-2-carboxamido)acetamido)-3,3-dimethylbutanoyl)octahydrocyclopenta[*c*]pyrrole-1-carboxylate (17 mg, 0.0178 mmol, 12%).
LC/MS *m*/*z* calculated for $[M+H]^+$ 954.5, found 954.5.

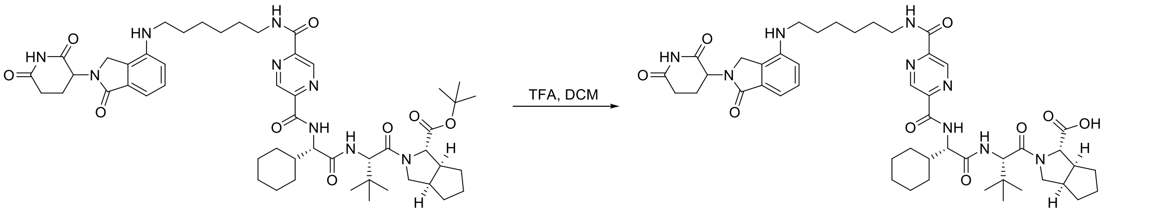

To the solution of (1*S*,3a*R*,6a*S*)-tert-butyl 2-((2*S*)-2-((2*S*)-2-cyclohexyl-2-(5-(6-(2-(2,6-dioxopiperidin-3-yl)-1-oxoisoindolin-4-ylamino)hexylcarbamoyl)pyrazine-2-carboxamido)acetamido)-3,3-dimethylbutanoyl)octahydrocyclopenta[*c*]pyrrole-1-carboxylate (17 mg, 0.0178 mmol) in DCM (1 mL) TFA (1 mL) was added at room temperature. The reaction was stirred for 1 hour. Removal of solvent under vacuum to obtain crude product (1*S*,3a*R*,6a*S*)-2-((2*S*)-2-((2*S*)-2-cyclohexyl-2-(5-(6-(2-(2,6-dioxopiperidin-3-yl)-1-oxoisoindolin-4-ylamino)hexylcarbamoyl)pyrazine-2-carboxamido)acetamido)-3,3-dimethylbutanoyl)octahydrocyclopenta[*c*]pyrrole-1-carboxylic acid (16 mg, 0.0178 mmol, 100%) without any further purification.
LC/MS *m*/*z* calculated for $[M+H]^+$ 898.5, found 898.5.

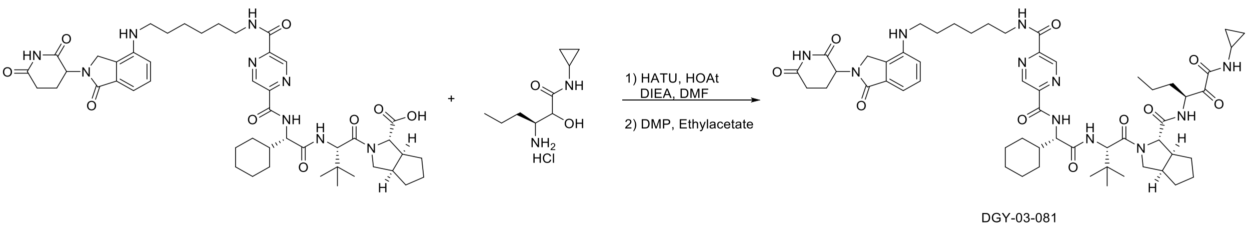

DGY-03-081

To a solution of (1*S*,3a*R*,6a*S*)-2-((2*S*)-2-((2*S*)-2-cyclohexyl-2-(5-(6-(2-(2,6-

dioxopiperidin-3-yl)-1-oxoisoindolin-4-ylamino)hexylcarbamoyl)pyrazine-2-carboxamido)acetamido)-3,3-dimethylbutanoyl)octahydrocyclopenta[c]pyrrole-1-carboxylic acid (16 mg, 0.0178 mmol) and (3*S*)-3-amino-*N*-cyclopropyl-2-hydroxyhexanamide HCl salt (4 mg, 0.0178 mmol) in DMF (1 mL), DIEA (12 mg, 0.089 mmol), HATU (13.5 mg, 0.0356 mmol) and HOAt (0.5 mg, 0.00356 mmol) were added under ice bath. The reaction was stirred for 10 mins. The solvent was evaporated under vacuum to get crude product without any further purification. The residue was dissolved in ethylacetate (1 mL). Then Dess Martin periodinane (10 mg, 0.023 mmol) was added to the mixture under ice bath. The reaction was stirred for 6 hours and purified with HPLC to get N^2-((*S*)-1-cyclohexyl-2-((*S*)-1-((1*S*,3a*R*,6a*S*)-1-((*S*)-1-(cyclopropylamino)-1,2-dioxohexan-3-ylcarbamoyl)hexahydrocyclopenta[*c*]pyrrol-2(1*H*)-yl)-3,3-dimethyl-1-oxobutan-2-ylamino)-2-oxoethyl)-N^5-(6-(2-(2,6-dioxopiperidin-3-yl)-1-oxoisoindolin-4-ylamino)hexyl)pyrazine-2,5-dicarboxamide (DGY-03-081, 3.6 mg, 0.0034 mmol, 19% by two steps). ^{1}H NMR (500 MHz, DMSO-*d6*) δ 11.00 (s, 1H), 9.21 (dd, *J* = 18.2, 1.5 Hz, 2H), 9.08 (t, *J* = 6.1 Hz, 1H), 8.70 (d, *J* = 5.2 Hz, 1H), 8.56 (d, *J* = 9.2 Hz, 1H), 8.23 (t, *J* = 10.4 Hz, 2H), 7.27 (t, *J* = 7.7 Hz, 1H), 6.92 (d, *J* = 7.4 Hz, 1H), 6.73 (d, *J* = 8.0 Hz, 1H), 5.11 (dd, *J* = 13.3, 5.1 Hz, 1H), 4.99 – 4.89 (m, 1H), 4.70 (dt, *J* = 9.1, 6.5 Hz, 1H), 4.53 (d, *J* = 9.1 Hz, 1H), 4.31 – 4.09 (m, 6H), 3.75 (t, *J* = 10.4 Hz, 1H), 3.64 (d, *J* = 10.6 Hz, 1H), 3.37 – 3.28 (m, 2H), 3.11 (t, *J* = 7.1 Hz, 2H), 2.98 – 2.86 (m, 1H), 2.78 – 2.69 (m, 1H), 2.68 – 2.57 (m, 2H), 2.37 – 2.22 (m, 1H), 2.08 – 1.97 (m, 1H), 1.87 – 1.27 (m, 24H), 1.19 – 0.99 (m, 4H), 0.94 (s, 9H), 0.91 – 0.83 (m, 3H), 0.71 – 0.61 (m, 2H), 0.60 – 0.51 (m, 2H). ^{13}C NMR (126 MHz, DMSO-d_6) δ 199.63, 175.55, 174.47, 173.90, 172.98, 171.64, 171.52, 164.75, 164.09, 149.68, 148.36, 146.38, 144.69, 144.45, 134.68, 131.84, 129.12, 114.37, 112.56, 67.37, 59.14, 59.06, 56.81, 56.07, 54.12, 49.86, 48.37, 45.32, 44.91, 43.90, 37.05, 34.73, 34.43, 34.28, 33.89, 31.69, 31.09, 30.72, 29.02, 28.36, 28.29, 28.22, 27.29, 25.47, 25.12, 21.41, 16.14, 8.06, 8.01. (These are diastereomers due to the CRBN binder materials are racemic) LC/MS *m*/*z* calculated for $[M+H]^+$ 1064.6, found 1064.6. HRMS calculated for $[M+H]^+$ 1064.5928, found 1064.5933.

DGY-04-035 synthesis and characterization

N^2-((*S*)-1-cyclohexyl-2-((*S*)-1-((1*S*,3a*R*,6a*S*)-1-((*S*)-1-(cyclopropylamino)-1,2-

dioxohexan-3-ylcarbamoyl)hexahydrocyclopenta[c]pyrrol-2(1*H*)-yl)-3,3-dimethyl-1-oxobutan-2-ylamino)-2-oxoethyl)-*N*[5]-(2-(2-(2-(2-(2,6-dioxopiperidin-3-yl)-1-oxoisoindolin-4-ylamino)ethoxy)ethoxy)ethyl)pyrazine-2,5-dicarboxamide (DGY-04-035).

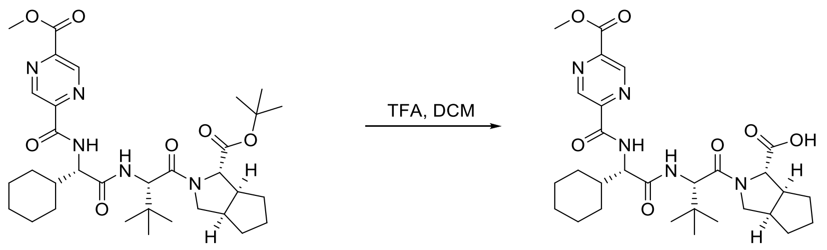

To the solution of (1*S*,3a*R*,6a*S*)-*tert*-butyl 2-((*S*)-2-((*S*)-2-cyclohexyl-2-(5-(methoxycarbonyl)pyrazine-2-carboxamido)acetamido)-3,3-dimethylbutanoyl)octahydrocyclopenta[*c*]pyrrole-1-carboxylate (560 mg, 0.89 mmol) in DCM (5 mL) TFA (2 mL) was added at room temperature. The reaction was stirred for 1 hour. Removal of solvent under vacuum to obtain crude product (1*S*,3a*R*,6a*S*)-2-((*S*)-2-((*S*)-2-cyclohexyl-2-(5-(methoxycarbonyl)pyrazine-2-carboxamido)acetamido)-3,3-dimethylbutanoyl)octahydrocyclopenta[*c*]pyrrole-1-carboxylic acid (510 mg, 0.89 mmol, 100%) without any further purification.
LC/MS *m*/*z* calculated for $[M+H]^+$ 572.3, found 572.3.

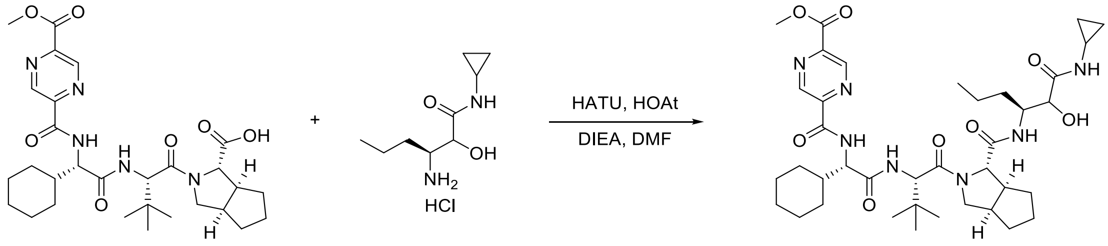

To a solution of (1*S*,3a*R*,6a*S*)-2-((*S*)-2-((*S*)-2-cyclohexyl-2-(5-(methoxycarbonyl)pyrazine-2-carboxamido)acetamido)-3,3-dimethylbutanoyl)octahydrocyclopenta[*c*]pyrrole-1-carboxylic acid (510 mg, 0.89 mmol) and (3*S*)-3-amino-*N*-cyclopropyl-2-hydroxyhexanamide HCl salt (198 mg, 0.89 mmol) in DMF (5 mL), DIEA (575 mg, 4.45 mmol), HATU (677 mg, 1.78 mmol) and HOAt (24 mg, 0.178 mmol) were added under ice bath. The reaction was stirred for 10 mins. Quenched the reaction with ice water. The mixture was stirred for 30 mins under ice bath. The white precipitate came out. Filtered the solid and washed

with ice water. Dried the solid under vacuum to get methyl 5-((1*S*)-1-cyclohexyl-2-((2*S*)-1-((1*S*,3a*R*,6a*S*)-1-((3*S*)-1-(cyclopropylamino)-2-hydroxy-1-oxohexan-3-ylcarbamoyl)hexahydrocyclopenta[*c*]pyrrol-2(1*H*)-yl)-3,3-dimethyl-1-oxobutan-2-ylamino)-2-oxoethylcarbamoyl)pyrazine-2-carboxylate (590 mg, 0.80 mmol, 90%). LC/MS *m*/*z* calculated for $[M+H]^+$ 740.4, found 740.4.

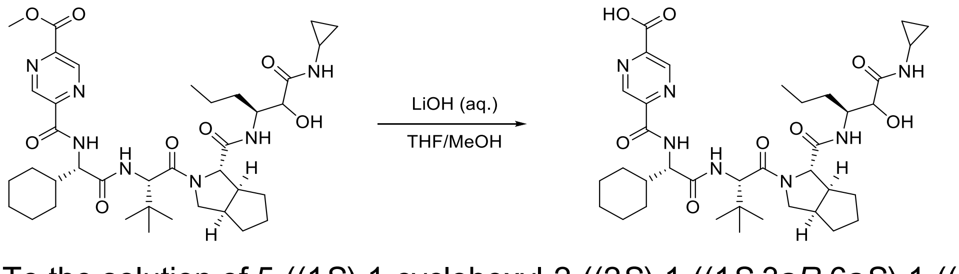

To the solution of 5-((1*S*)-1-cyclohexyl-2-((2*S*)-1-((1*S*,3a*R*,6a*S*)-1-((3*S*)-1-(cyclopropylamino)-2-hydroxy-1-oxohexan-3-ylcarbamoyl)hexahydrocyclopenta[*c*]pyrrol-2(1*H*)-yl)-3,3-dimethyl-1-oxobutan-2-ylamino)-2-oxoethylcarbamoyl)pyrazine-2-carboxylate (590 mg, 0.80 mmol) in THF/MeOH (5 mL/0.3 mL) LiOH (aq., 1.6 mL, 1.60 mmol, 1M) was added at room temperature. The reaction was stirred overnight. To the mixture, HCl (aq. 2M) was added to acidify the solution to pH = 3. Extracted the mixture with EA (20 mL * 3). The organic layer was combined and evaporated under vacuum to get crude product 5-((1*S*)-1-cyclohexyl-2-((2*S*)-1-((1*S*,3a*R*,6a*S*)-1-((3*S*)-1-(cyclopropylamino)-2-hydroxy-1-oxohexan-3-ylcarbamoyl)hexahydrocyclopenta[*c*]pyrrol-2(1*H*)-yl)-3,3-dimethyl-1-oxobutan-2-ylamino)-2-oxoethylcarbamoyl)pyrazine-2-carboxylic acid (568 mg, 0.784 mmol, 98%) without any further purification.
LC/MS *m*/*z* calculated for $[M+H]^+$ 726.4, found 726.4.

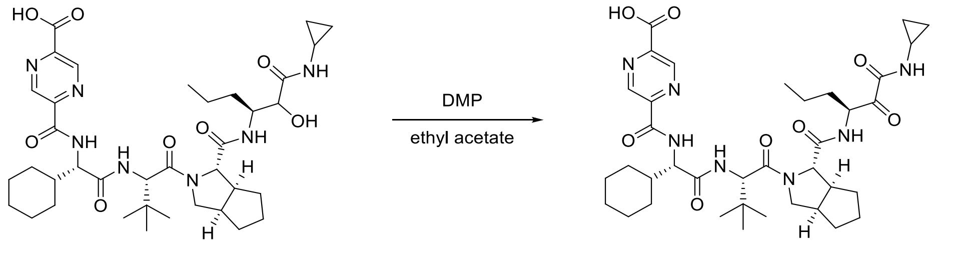

To the solution of 5-((1*S*)-1-cyclohexyl-2-((2*S*)-1-((1*S*,3a*R*,6a*S*)-1-((3*S*)-1-(cyclopropylamino)-2-hydroxy-1-oxohexan-3-ylcarbamoyl)hexahydrocyclopenta[*c*]pyrrol-2(1*H*)-yl)-3,3-dimethyl-1-oxobutan-2-

ylamino)-2-oxoethylcarbamoyl)pyrazine-2-carboxylic acid (20 mg, 0.027 mmol) in ethyl acetate (1 mL), DMP (23.4 mg, 0.055 mmol) was added under ice bath. The reaction mixture was stirred under ice bath for 6 hours. Filtered the reaction with celite and concentrated the filtrate under vacuum. The residue was purified with ISCO to get 5-((*S*)-1-cyclohexyl-2-((*S*)-1-((1*S*,3a*R*,6a*S*)-1-((*S*)-1-(cyclopropylamino)-1,2-dioxohexan-3-ylcarbamoyl)hexahydrocyclopenta[*c*]pyrrol-2(1*H*)-yl)-3,3-dimethyl-1-oxobutan-2-ylamino)-2-oxoethylcarbamoyl)pyrazine-2-carboxylic acid (13 mg, 0.018 mmol, 67%).

LC/MS m/z calculated for [M+H]+ 724.4, found 724.4.

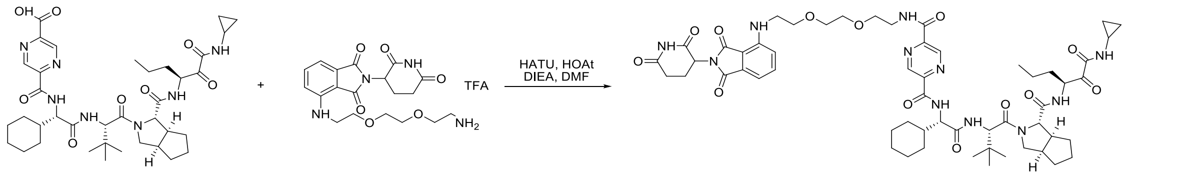

DGY-04-035

To a solution of 5-((*S*)-1-cyclohexyl-2-((*S*)-1-((1*S*,3a*R*,6a*S*)-1-((*S*)-1-(cyclopropylamino)-1,2-dioxohexan-3-ylcarbamoyl)hexahydrocyclopenta[*c*]pyrrol-2(1*H*)-yl)-3,3-dimethyl-1-oxobutan-2-ylamino)-2-oxoethylcarbamoyl)pyrazine-2-carboxylic acid (10 mg, 0.0138 mmol) and 4-(2-(2-(2-aminoethoxy)ethoxy)ethylamino)-2-(2,6-dioxopiperidin-3-yl)isoindoline-1,3-dione TFA salt (7 mg, 0.0138 mmol) in DMF (1 mL), DIEA (9 mg, 0.069 mmol), HATU (10.5 mg, 0.0276 mmol) and HOAt (0.4 mg, 0.00276 mmol) were added under ice bath. The reaction was stirred for 10 mins. The reaction mixture was purified with HPLC to get N^2-((*S*)-1-cyclohexyl-2-((*S*)-1-((1*S*,3a*R*,6a*S*)-1-((*S*)-1-(cyclopropylamino)-1,2-dioxohexan-3-ylcarbamoyl)hexahydrocyclopenta[c]pyrrol-2(1*H*)-yl)-3,3-dimethyl-1-oxobutan-2-ylamino)-2-oxoethyl)-N^5-(2-(2-(2-(2-(2,6-dioxopiperidin-3-yl)-1,3-dioxoisoindolin-4-ylamino)ethoxy)ethoxy)ethyl)pyrazine-2,5-dicarboxamide (DGY-04-035, 12 mg, 0.0108 mmol, 78%). δ^{1}H NMR (500 MHz, DMSO-d_6) δ 11.08 (s, 1H), 9.21 (s, 1H), 9.15 (s, 1H), 8.90 (t, *J* = 5.9 Hz, 1H), 8.70 (d, *J* = 5.2 Hz, 1H), 8.55 (d, *J* = 9.1 Hz, 1H), 8.23 (t, *J* = 8.6 Hz, 2H), 7.54 (t, *J* = 7.1 Hz, 1H), 7.09 (d, *J* = 8.6 Hz, 1H), 7.00 (d, *J* = 7.1 Hz, 1H), 5.05 (dd, *J* = 12.9, 5.4 Hz, 1H), 5.01 – 4.89 (m, 1H), 4.69 (d, *J* = 9.1, Hz, 1H), 4.54 (d, *J* = 9.1 Hz, 1H), 4.27 (d, *J* = 3.4 Hz, 1H), 3.75 (dd, *J* = 10.4, 7.6 Hz, 1H), 3.67 – 3.61 (m, 3H), 3.61 – 3.55 (m, 6H), 3.53 – 3.46 (m, 2H), 3.46 – 3.40 (m, 2H), 2.94 – 2.82 (m, 1H), 2.78 – 2.69 (m, 1H), 2.67 – 2.58 (m, 2H),

2.58 – 2.52 (m, 2H), 2.08 – 1.99 (m, 1H), 1.86 – 1.30 (m, 18H), 1.20 – 1.00 (m, 4H), 0.94 (s, 9H), 0.91 – 0.82 (m, 3H), 0.68 – 0.60 (m, 2H), 0.60 – 0.51 (m, 2H). ^{13}C NMR (126 MHz, DMSO-d_6) δ 199.55, 175.53, 174.51, 173.04, 172.71, 171.67, 171.52, 169.90, 164.85, 164.78, 164.09, 161.08, 160.80, 149.17, 148.93, 148.40, 144.54, 144.46, 138.82, 134.58, 119.97, 113.29, 111.73, 72.25, 72.19, 71.42, 71.16, 67.40, 59.24, 59.06, 56.80, 56.04, 51.22, 49.82, 44.89, 44.30, 43.78, 37.06, 34.70, 34.38, 34.20, 33.58, 31.65, 30.73, 28.95, 28.30, 28.23, 28.17, 27.24, 25.09, 24.74, 21.37, 16.08, 8.06, 8.01. (These are diastereomers due to the CRBN binder materials are racemic) LC/MS *m/z* calculated for $[M+H]^+$ 1110.6, found 1110.6. HRMS calculated for $[M+H]^+$ 1110.5619, found 1110.5615.

DGY-08-097 synthesis and characterization

<u>*N*^{2}-((*S*)-1-cyclohexyl-2-((*S*)-1-((1*S*,3a*R*,6a*S*)-1-((*S*)-1-(cyclopropylamino)-1,2-dioxohexan-3-ylcarbamoyl)hexahydrocyclopenta[*c*]pyrrol-2(1*H*)-yl)-3,3-dimethyl-1-oxobutan-2-ylamino)-2-oxoethyl)-*N*^{5}-(2-(2-(2-(2-(2,6-dioxopiperidin-3-yl)-1,3-dioxo-2,3-dihydro-1*H*-benzo[*de*]isoquinolin-5-yloxy)ethoxy)ethoxy)ethyl)pyrazine-2,5-dicarboxamide (DGY-08-097).</u>

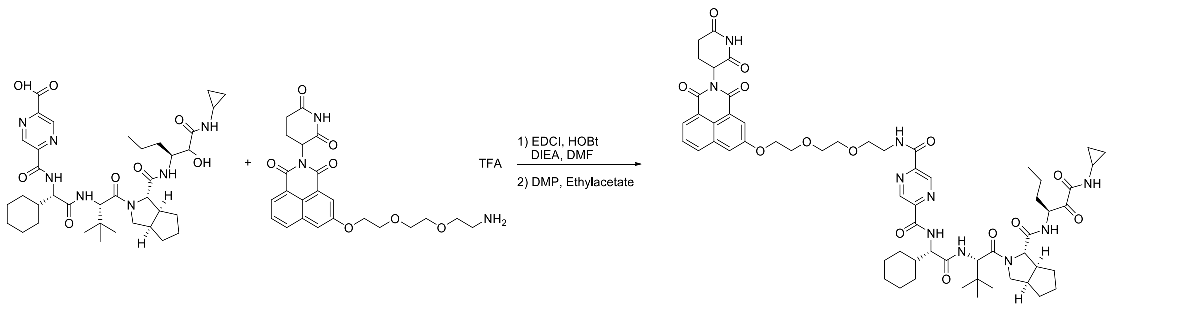

DGY-08-097

To a solution of 5-((1*S*)-1-cyclohexyl-2-((2*S*)-1-((1*S*,3a*R*,6a*S*)-1-((3*S*)-1-(cyclopropylamino)-2-hydroxy-1-oxohexan-3-ylcarbamoyl)hexahydrocyclopenta[*c*]pyrrol-2(1*H*)-yl)-3,3-dimethyl-1-oxobutan-2-ylamino)-2-oxoethylcarbamoyl)pyrazine-2-carboxylic acid (29 mg, 0.039 mmol) and 5-(5-aminopentyloxy)-2-(2,6-dioxopiperidin-3-yl)-1*H*-benzo[*de*]isoquinoline-1,3(2*H*)-dioneTFA salt (18 mg, 0.039 mmol) in DMF (1 mL), DIEA (25 mg, 0.195 mmol), EDCI (15 mg, 0.078 mmol) and HOBt (11 mg, 0.078 mmol) were added under ice bath. The reaction was stirred for overnight. The solvent was evaporated under vacuum to get crude product without any further purification. The residue was

dissolved in ethyl acetate (1 mL). Then Dess Martin periodinane (29 mg, 0.069 mmol) was added to the mixture under ice bath. The reaction was stirred for 6 hours and purified with HPLC to get N^2-((*S*)-1-cyclohexyl-2-((*S*)-1-((1*S*,3a*R*,6a*S*)-1-((*S*)-1-(cyclopropylamino)-1,2-dioxohexan-3-ylcarbamoyl)hexahydrocyclopenta[*c*]pyrrol-2(1*H*)-yl)-3,3-dimethyl-1-oxobutan-2-ylamino)-2-oxoethyl)-N^5-(2-(2-(2-(2-(2,6-dioxopiperidin-3-yl)-1,3-dioxo-2,3-dihydro-1*H*-benzo[*de*]isoquinolin-5-yloxy)ethoxy)ethoxy)ethyl)pyrazine-2,5-dicarboxamide (DGY-08-097, 3.6 mg, 0.0031 mmol, 8% by two steps). ^{1}H NMR (500 MHz, DMSO-d_6) δ 10.94 (s, 1H), 9.17 – 9.11 (m, 1H), 9.10 – 9.06 (m, 1H), 8.89 – 8.80 (m, 1H), 8.63 (d, *J* = 5.2 Hz, 1H), 8.52 – 8.41 (m, 1H), 8.36 – 8.10 (m, 4H), 7.98 (dt, *J* = 49.6, 2.8 Hz, 1H), 7.90 (d, *J* = 2.6 Hz, 1H), 7.75 (q, *J* = 8.6, 7.9 Hz, 1H), 5.81 – 5.69 (m, 1H), 4.93 – 4.83 (m, 1H), 4.61 (t, *J* = 7.1 Hz, 1H), 4.46 (d, *J* = 9.1 Hz, 1H), 4.28 (d, *J* = 4.7 Hz, 2H), 4.20 (d, *J* = 3.4 Hz, 1H), 3.85 – 3.75 (m, 2H), 3.68 (dd, *J* = 10.4, 7.7 Hz, 1H), 3.62 – 3.47 (m, 6H), 3.48 – 3.38 (m, 2H), 2.96 – 2.81 (m, 1H), 2.73 – 2.62 (m, 2H), 2.61 – 2.49 (m, 3H), 2.04 – 1.94 (m, 1H), 1.81 – 1.23 (m, 18H), 1.12 – 0.92 (m, 4H), 0.87 (s, 9H), 0.82 – 0.75 (m, 3H), 0.63 – 0.56 (m, 2H), 0.55 – 0.46 (m, 2H). ^{13}C NMR (126 MHz, DMSO-d_6) δ 199.53, 175.61, 174.52, 173.05, 172.89, 171.68, 166.07, 165.65, 165.29, 164.88, 164.79, 164.10, 159.62, 149.13, 148.34, 144.45, 136.22, 136.16, 135.67, 131.61, 131.01, 130.46, 130.40, 125.82, 125.46, 125.37, 125.28, 124.62, 124.22, 123.85, 117.34, 117.27, 72.55, 72.14, 71.32, 71.12, 70.79, 67.41, 59.28, 59.06, 56.80, 56.03, 53.16, 49.81, 44.88, 43.72, 37.06, 34.69, 34.36, 34.18, 33.42, 31.63, 31.56, 30.72, 28.93, 28.28, 28.22, 28.15, 27.23, 25.09, 24.00, 21.36, 16.07, 8.06, 8.02. (These are diastereomers due to the CRBN binder materials are racemic) LC/MS *m*/*z* calculated for $[M+H]^+$ 1161.5, found 1161.5. HRMS calculated for $[M+H]^+$ 1161.5615, found 1161.5613.

DGY-04-035-BUMP synthesis and characterization

N^2-((*S*)-1-cyclohexyl-2-((*S*)-1-((1*S*,3a*R*,6a*S*)-1-((*S*)-1-(cyclopropylamino)-1,2-dioxohexan-3-ylcarbamoyl)hexahydrocyclopenta[*c*]pyrrol-2(1*H*)-yl)-3,3-dimethyl-1-oxobutan-2-ylamino)-2-oxoethyl)-N^5-(2-(2-(2-(2-(1-methyl-2,6-dioxopiperidin-3-yl)-1,3-dioxoisoindolin-4-ylamino)ethoxy)ethoxy)ethyl)pyrazine-2,5-dicarboxamide (DGY-04-035-BUMP).

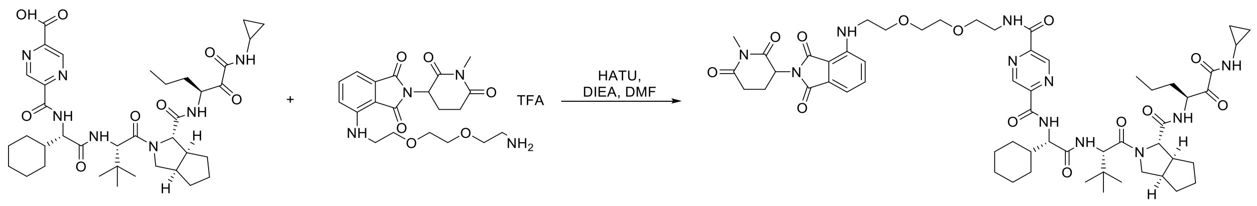

DGY-04-035-BUMP

To a solution of 5-((*S*)-1-cyclohexyl-2-((*S*)-1-((1*S*,3a*R*,6a*S*)-1-((*S*)-1-(cyclopropylamino)-1,2-dioxohexan-3-ylcarbamoyl)hexahydrocyclopenta[*c*]pyrrol-2(1*H*)-yl)-3,3-dimethyl-1-oxobutan-2-ylamino)-2-oxoethylcarbamoyl)pyrazine-2-carboxylic acid (20 mg, 0.028 mmol) and 4-(2-(2-(2-aminoethoxy)ethoxy)ethylamino)-2-(1-methyl-2,6-dioxopiperidin-3-yl)isoindoline-1,3-dione TFA salt (15 mg, 0.028 mmol) in DMF (1 mL), DIEA (18 mg, 0.14 mmol) and HATU (21 mg, 0.055 mmol) were added under ice bath. The reaction was stirred for 10 mins. The reaction mixture was purified with HPLC to get N^2-((*S*)-1-cyclohexyl-2-((*S*)-1-((1*S*,3a*R*,6a*S*)-1-((*S*)-1-(cyclopropylamino)-1,2-dioxohexan-3-ylcarbamoyl)hexahydrocyclopenta[c]pyrrol-2(1*H*)-yl)-3,3-dimethyl-1-oxobutan-2-ylamino)-2-oxoethyl)-N^5-(2-(2-(2-(2-(1-methyl-2,6-dioxopiperidin-3-yl)-1,3-dioxoisoindolin-4-ylamino)ethoxy)ethoxy)ethyl)pyrazine-2,5-dicarboxamide (DGY-04-035-BUMP, 12 mg, 0.0107 mmol, 38%). ^{1}H NMR (500 MHz, DMSO-d_6) δ 9.21 (d, *J* = 1.4 Hz, 1H), 9.15 (d, *J* = 1.4 Hz, 1H), 8.89 (t, *J* = 5.9 Hz, 1H), 8.71 (d, *J* = 5.2 Hz, 1H), 8.55 (d, *J* = 9.0 Hz, 1H), 8.23 (t, *J* = 8.7 Hz, 2H), 7.55 (dd, *J* = 8.5, 7.1 Hz, 1H), 7.16 – 7.08 (m, 1H), 7.05 – 6.98 (m, 1H), 5.12 (dd, *J* = 13.0, 5.4 Hz, 1H), 4.99 – 4.91 (m, 1H), 4.70 (dd, *J* = 9.1, 6.5 Hz, 1H), 4.54 (d, *J* = 9.1 Hz, 1H), 4.28 (d, *J* = 3.4 Hz, 1H), 3.81 – 3.71 (m, 1H), 3.69 – 3.62 (m, 3H), 3.62 – 3.55 (m, 6H), 3.53 – 3.41 (m, 4H), 3.00 (s, 3H), 2.99 – 2.89 (m, 1H), 2.79 – 2.71 (m, 2H), 2.68 – 2.62 (m, 1H), 2.61 – 2.53 (m, 2H), 2.12 – 2.01 (m, 1H), 1.87 – 1.31 (m, 18H), 1.22 – 1.01 (m, 4H), 0.94 (s, 9H), 0.91 – 0.85 (m, 3H), 0.69 – 0.63 (m, 2H), 0.61 – 0.52 (m, 2H); ^{13}C NMR (126 MHz, DMSO-d_6) δ 199.64, 174.49, 174.44, 173.00, 172.46, 171.66, 171.55, 169.87, 164.81, 164.77, 164.04, 161.01, 160.73, 149.26, 149.01, 148.48, 144.62, 144.48, 138.83, 134.69, 120.04, 113.30, 111.83, 72.32, 72.21, 71.49, 71.23, 67.39, 59.16, 59.09, 56.83, 56.09, 51.78, 49.88, 44.93, 44.35, 43.92, 37.07, 34.74, 34.44, 34.30, 33.80, 31.71, 30.73, 29.24, 29.02, 28.37, 28.30, 28.24, 27.30, 25.13, 24.00, 21.42, 16.15, 8.07, 8.02. (These are diastereomers due to the CRBN binder materials are racemic) LC/MS *m*/*z* calculated for $[M+H]^+$ 1124.6, found 1124.6. HRMS calculated

for [M+H]$^+$ 1124.5775, found 1124.5770.

DGY-08-097-BUMP synthesis and characterization

N^2-((*S*)-1-cyclohexyl-2-((*S*)-1-((1*S*,3a*R*,6a*S*)-1-((*S*)-1-(cyclopropylamino)-1,2-dioxohexan-3-ylcarbamoyl)hexahydrocyclopenta[*c*]pyrrol-2(1*H*)-yl)-3,3-dimethyl-1-oxobutan-2-ylamino)-2-oxoethyl)-*N*^{5}-(2-(2-(2-(2-(1-methyl-2,6-dioxopiperidin-3-yl)-1,3-dioxo-2,3-dihydro-1*H*-benzo[*de*]isoquinolin-5-yloxy)ethoxy)ethoxy)ethyl)pyrazine-2,5-dicarboxamide (DGY-08-097-BUMP).

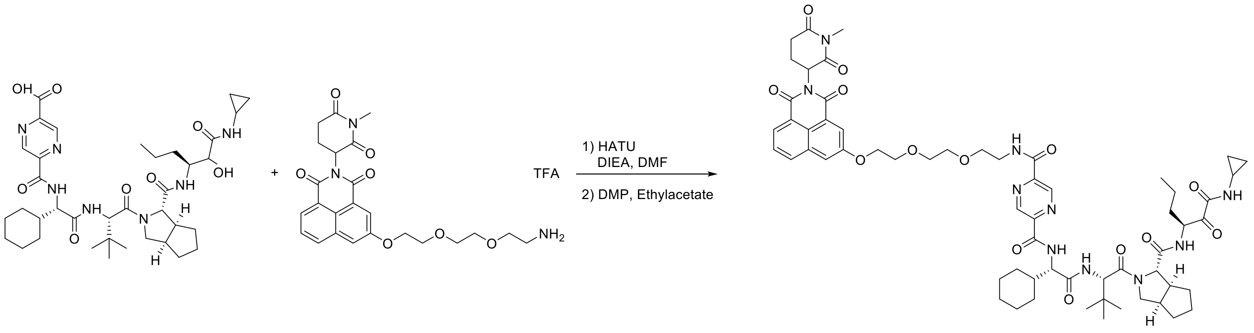

DGY-08-097-BUMP

To a solution of 5-((1*S*)-1-cyclohexyl-2-((2*S*)-1-((1*S*,3a*R*,6a*S*)-1-((3*S*)-1-(cyclopropylamino)-2-hydroxy-1-oxohexan-3-ylcarbamoyl)hexahydrocyclopenta[*c*]pyrrol-2(1*H*)-yl)-3,3-dimethyl-1-oxobutan-2-ylamino)-2-oxoethylcarbamoyl)pyrazine-2-carboxylic acid (20 mg, 0.027 mmol) and 5-(2-(2-(2-aminoethoxy)ethoxy)ethoxy)-2-(1-methyl-2,6-dioxopiperidin-3-yl)-1*H*-benzo[*de*]isoquinoline-1,3(2*H*)-dione TFA salt (16 mg, 0.027 mmol) in DMF (1 mL), DIEA (18 mg, 0.138 mmol) and HATU (21 mg, 0.054 mmol) were added under ice bath. The reaction was stirred for 10 min. The solvent was evaporated under vacuum to get crude product without any further purification. The residue was dissolved in ethyl acetate (1 mL). Then Dess Martin periodinane (32 mg, 0.076 mmol) was added to the mixture under ice bath. The reaction was stirred for 6 hours and purified with HPLC to get *N*^{2}-((*S*)-1-cyclohexyl-2-((*S*)-1-((1*S*,3a*R*,6a*S*)-1-((*S*)-1-(cyclopropylamino)-1,2-dioxohexan-3-ylcarbamoyl)hexahydrocyclopenta[*c*]pyrrol-2(1*H*)-yl)-3,3-dimethyl-1-oxobutan-2-ylamino)-2-oxoethyl)-*N*^{5}-(2-(2-(2-(2-(1-methyl-2,6-dioxopiperidin-3-yl)-1,3-dioxo-2,3-dihydro-1*H*-benzo[*de*]isoquinolin-5-yloxy)ethoxy)ethoxy)ethyl)pyrazine-2,5-dicarboxamide (DGY-08-097-BUMP, 6.7 mg, 0.0057 mmol, 21% by two steps). ^{1}H NMR (500 MHz, DMSO-d_6) δ 9.16 – 9.04 (m, 2H), 8.85 (t, *J* = 5.8 Hz, 1H), 8.63 (d, *J* = 5.2 Hz, 1H), 8.46 (d, *J* = 8.8 Hz, 1H), 8.36 –

8.09 (m, 4H), 7.99 (dt, *J* = 51.2, 3.2 Hz, 1H), 7.91 (d, *J* = 2.6 Hz, 1H), 7.75 (dt, *J* = 15.6, 8.0 Hz, 1H), 5.85 (dt, *J* = 12.6, 6.3 Hz, 1H), 4.91 – 4.82 (m, 1H), 4.65 – 4.58 (m, 1H), 4.46 (d, *J* = 9.1 Hz, 1H), 4.31 – 4.23 (m, 2H), 4.20 (d, *J* = 3.3 Hz, 1H), 3.83 – 3.74 (m, 2H), 3.72 – 3.65 (m, 1H), 3.62 – 3.29 (m, 8H), 2.98 (s, 3H), 2.97 – 2.87 (m, 1H), 2.74 – 2.63 (m, 2H), 2.61 – 2.46 (m, 3H), 2.04 – 1.95 (m, 1H), 1.79 – 1.22 (m, 18H), 1.11 – 0.90 (m, 4H), 0.86 (s, 9H), 0.84 – 0.77 (m, 3H), 0.59 – 0.54 (m, 2H), 0.52 – 0.46 (m, 2H); ^{13}C NMR (126 MHz, DMSO-d_6) δ 199.52, 174.65, 174.53, 173.06, 172.64, 171.68, 165.98, 165.55, 165.35, 164.88, 164.80, 164.11, 159.62, 149.10, 148.32, 144.45, 136.21, 136.15, 135.66, 131.68, 131.09, 130.46, 130.40, 125.75, 125.42, 125.37, 125.32124.62, 124.14, 123.81, 117.51, 117.33, 72.55, 72.13, 71.32, 71.11, 70.81, 67.42, 59.29, 59.06, 57.39, 56.80, 56.03, 53.70, 49.80, 44.88, 43.71, 37.06, 34.69, 34.36, 34.17, 33.54, 31.62, 30.72, 29.16, 28.92, 28.27, 28.21, 28.14, 27.23, 25.09, 23.25, 21.35, 16.06, 8.06, 8.02. (These are diastereomers due to the CRBN binder materials are racemic) LC/MS *m*/*z* calculated for $[M+H]^+$ 1175.6, found 1175.6. HRMS calculated for $[M+H]^+$ 1175.5772, found 1175.5757.

SUPPLEMENTARY REFERENCES

1 Nowak, R. P. *et al.* Plasticity in binding confers selectivity in ligand-induced protein degradation. *Nat Chem Biol*, doi:10.1038/s41589-018-0055-y (2018).

2 Ritchie, M. E. *et al.* limma powers differential expression analyses for RNA-sequencing and microarray studies. *Nucleic Acids Res* **43**, e47, doi:10.1093/nar/gkv007 (2015).
