# Supplementary material for: Small molecule degraders of the hepatitis C virus protease reduce susceptibility to resistance mutations
Source: Nat Commun. 2019 Aug 1;10:3468. doi: 10.1038/s41467-019-11429-w (PMC6672008; doi:10.1038/s41467-019-11429-w)
Supplement: Supplementary file 3 — Reporting Summary [file 41467_2019_11429_MOESM3_ESM.pdf]

nature research

Corresponding author(s): Priscilla L. Yang

Reporting Summary

Nature Research wishes to improve the reproducibility of the work that we publish. This form provides structure for consistency and transparency in reporting. For further information on Nature Research policies, see Authors & Referees and the Editorial Policy Checklist.

Statistical parameters

When statistical analyses are reported, confirm that the following items are present in the relevant location (e.g. figure legend, table legend, main text, or Methods section).

n/a	Confirmed	
☐	☒	The exact sample size (*n*) for each experimental group/condition, given as a discrete number and unit of measurement
☐	☒	An indication of whether measurements were taken from distinct samples or whether the same sample was measured repeatedly
☐	☒	The statistical test(s) used AND whether they are one- or two-sided<br>*Only common tests should be described solely by name; describe more complex techniques in the Methods section.*
☒	☐	A description of all covariates tested
☒	☐	A description of any assumptions or corrections, such as tests of normality and adjustment for multiple comparisons
☐	☒	A full description of the statistics including central tendency (e.g. means) or other basic estimates (e.g. regression coefficient) AND variation (e.g. standard deviation) or associated estimates of uncertainty (e.g. confidence intervals)
☐	☒	For null hypothesis testing, the test statistic (e.g. *F*, *t*, *r*) with confidence intervals, effect sizes, degrees of freedom and *P* value noted<br>*Give P values as exact values whenever suitable.*
☒	☐	For Bayesian analysis, information on the choice of priors and Markov chain Monte Carlo settings
☒	☐	For hierarchical and complex designs, identification of the appropriate level for tests and full reporting of outcomes
☒	☐	Estimates of effect sizes (e.g. Cohen's *d*, Pearson's *r*), indicating how they were calculated
☐	☒	Clearly defined error bars<br>*State explicitly what error bars represent (e.g. SD, SE, CI)*

Our web collection on statistics for biologists may be useful.

Software and code

Policy information about availability of computer code

Data collection	BD FACSDiva Software
Data analysis	GraphPad Prism 6, FlowJo 10.3, CellProfiler, Proteome Discoverer 2.2, R framework, ImageJ

For manuscripts utilizing custom algorithms or software that are central to the research but not yet described in published literature, software must be made available to editors/reviewers upon request. We strongly encourage code deposition in a community repository (e.g. GitHub). See the Nature Research guidelines for submitting code & software for further information.

Data

Policy information about availability of data

All manuscripts must include a data availability statement. This statement should provide the following information, where applicable:

- Accession codes, unique identifiers, or web links for publicly available datasets
- A list of figures that have associated raw data
- A description of any restrictions on data availability

Mass spectrometry raw data files will be deposited in PRIDE Archive under the data set identifier PXD014346.
The authors declare that the data supporting the findings of this study are available within the paper and its supplementary information files. Any additional data are available from the corresponding author upon reasonable request.

Field-specific reporting

Please select the best fit for your research. If you are not sure, read the appropriate sections before making your selection.

☒ Life sciences ☐ Behavioural & social sciences ☐ Ecological, evolutionary & environmental sciences

For a reference copy of the document with all sections, see nature.com/authors/policies/ReportingSummary-flat.pdf

Life sciences study design

All studies must disclose on these points even when the disclosure is negative.

Sample size	Samples sizes are described in the figures legends. Where indicated, means from 2 to 4 technical replicates were calculated. All biological and biochemical experiments were performed independently at least 2 times. To determine inhibitory or effective concentrations values (IC50, EC50, DC50, CC50), the means from 2 to 5 independently performed experiments were calculated.
Data exclusions	No data exclusions.
Replication	Findings were successfully reproduced.
Randomization	In experiments involving cultured cells, low passage populations were distributed into multi-well plates one day prior treatment. All other experiments would not benefit from randomization.
Blinding	The study did not involve animals or human subjects. The investigators were not blinded during analysis, as all results are quantitative or semi-quantitative and would not benefit from blinding.

Reporting for specific materials, systems and methods

Materials & experimental systems

n/a	Involved in the study
☐	☒ Unique biological materials
☐	☒ Antibodies
☐	☒ Eukaryotic cell lines
☒	☐ Palaeontology
☒	☐ Animals and other organisms
☒	☐ Human research participants

Methods

n/a	Involved in the study
☒	☐ ChIP-seq
☐	☒ Flow cytometry
☒	☐ MRI-based neuroimaging

Unique biological materials

Policy information about availability of materials

Obtaining unique materials	No restrictions for academic users.

Antibodies

Antibodies used	Mouse monoclonal antibody 9E10 anti-HCV NS5A was provided by Charles Rice (Rockefeller University).

	Other antibodies were purchased from the following vendors:<br>Mouse monoclonal antibody anti-HCV NS3: Abcam ab65407 (RRID:AB_1139514).<br>Mouse monoclonal antibody against GAPDH: GeneTex GTX28245 (RRID:AB_370675)<br>Rabbit polyclonal antibody against CRBN: Novus Biologicals NBP1-91810 (RRID:AB_11037820)<br>Rabbit polyclonal antibody against SOD1: Sigma-Aldrich HPA00140-1 (RRID:AB_1080132)<br>Horseradish peroxidase (HRP)-conjugated goat anti-mouse IgG antibody: Bio-Rad Laboratories 170-6516 (RRID:AB_11125547)<br>Horseradish peroxidase (HRP)-conjugated goat anti-rabbit IgG antibody: Bio-Rad Laboratories 170-6515 (RRID:AB_11125142)<br>IRDye® 800CW-conjugated goat anti-rabbit antibody: LI-COR 926-32211 (RRID:AB_621843)
Validation	Mouse monoclonal antibody 9E10 anti-HCV NS5A was validated in Lindenbach et al., 2005.<br>All commercial antibodies were validated for the indicated use by the manufacturer, and validation is available on their website.<br>Mouse monoclonal antibody anti-HCV NS3 was additionally validated in house and showed no cross-reactivity for any host protein when tested against mock-infected samples that did not contain HCV.

Eukaryotic cell lines

Policy information about cell lines

Cell line source(s)	Huh7.5 cells (RRID:CVCL_7927) were obtained from Charles Rice (Rockefeller University).<br>Flp-In T-REx 293 cells (RRID:CVCL_U427) were purchased from Thermo Fisher Scientific (R78007).<br>Flp-In 293 cells (RRID:CVCL_U421) were purchased from Thermo Fisher Scientific (R75007)<br>HEK293T cells (RRID:CVCL_0063) were purchased from ATCC (CRL-3216)
Authentication	None of the cell lines were authenticated.
Mycoplasma contamination	All cell lines were routinely checked for mycoplasma contamination using the LookOut® Mycoplasma PCR Detection Kit (Sigma).
Commonly misidentified lines<br>(See ICLAC register)	Not applicable.

Flow Cytometry

Plots

Confirm that:

☒ The axis labels state the marker and fluorochrome used (e.g. CD4-FITC).

☒ The axis scales are clearly visible. Include numbers along axes only for bottom left plot of group (a 'group' is an analysis of identical markers).

☒ All plots are contour plots with outliers or pseudocolor plots.

☒ A numerical value for number of cells or percentage (with statistics) is provided.

Methodology

Sample preparation	Flp-In T-REx 293 stable cells expressing the HCV NS3-eGFP protein fusion and the mCherry reporter were lifted with Versene (Thermo Fisher Scientific 15040-066) and resuspended in PBS.
Instrument	The cells were analyzed using a BDTM LSR II flow cytometer (BD Biosciences).
Software	Data were analyzed using FlowJo (FlowJo, LCC).
Cell population abundance	Signal from at least 10,000 events per sample was acquired.
Gating strategy	Forward and side scatter outliers, frequently associated with cell debris, were removed leaving > 90% of total cells. The eGFP and mCherry fluorescence was monitored and positive fluorescence boundaries were determined by measuring the fluorescence from non-induced samples that do not express the protein fusion. After removal of eGFP and mCherry signal outliers, 55–85% of total cells were left and used for quantification.

☒ Tick this box to confirm that a figure exemplifying the gating strategy is provided in the Supplementary Information.
